# Supplementary material for: Optimizing synthetic cystic fibrosis sputum media for growth of non-typeable Haemophilus influenzae
Source: Access Microbiol. 2025 Jun 20;7(6):000979.v3. doi: 10.1099/acmi.0.000979.v3 (PMC12181625; doi:10.1099/acmi.0.000979.v3)
Supplement: Uncited Supplementary Material 1. [file acmi-7-00979-s001.pdf]

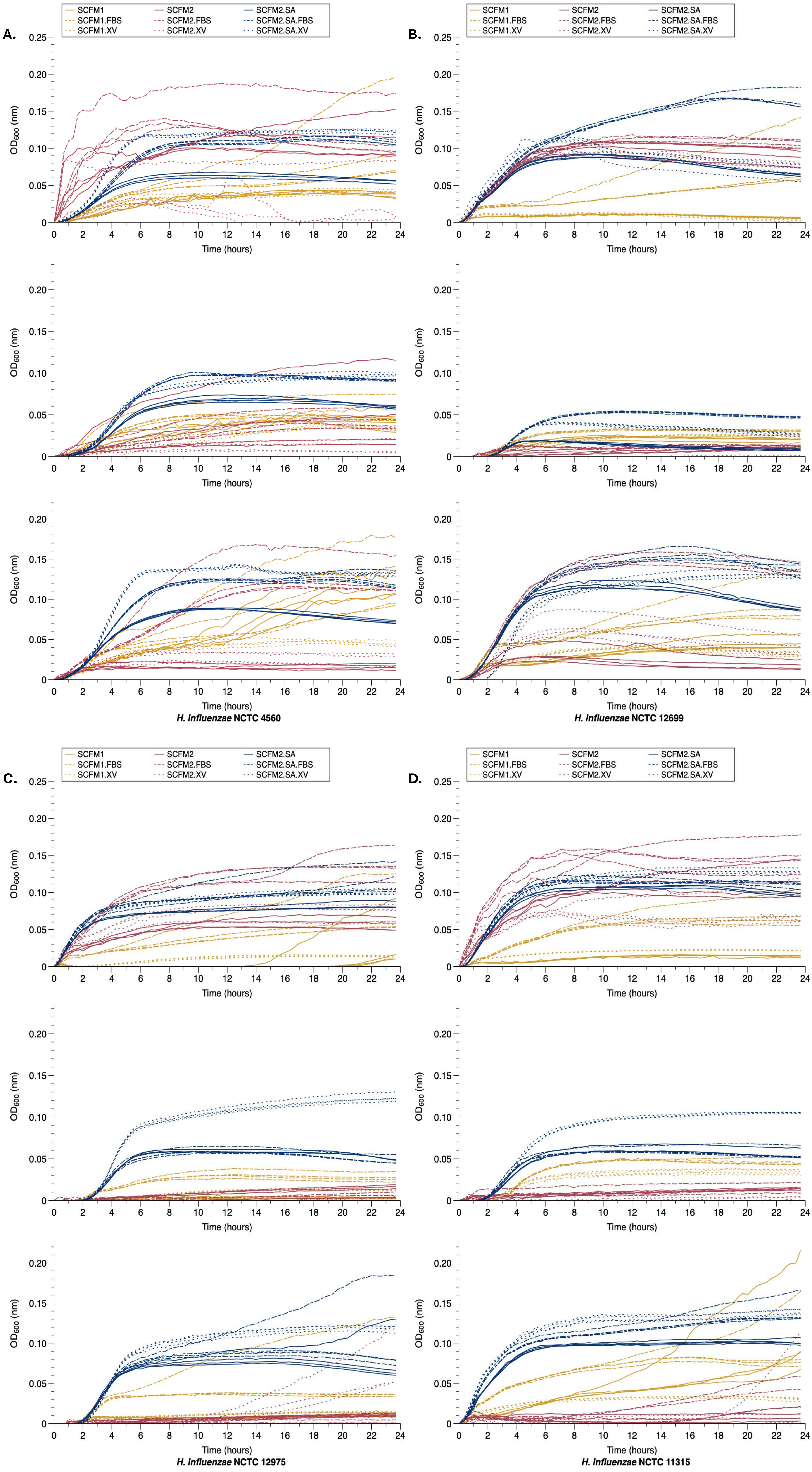

Supplementary Figure 1: Growth of *H. influenzae* laboratory strains in variants of SCFM over 24 hours. Three independent repeats were performed for each lab strain, **A.** *H. influenzae* NCTC 4560, **B.** *H. influenzae* NCTC 12699, **C.** *H. influenzae* NCTC 12975 and **D.** *H. influenzae* NCTC 11315 in 9 variants of media, SCFM1, SCFM2 and modified SCFM2 with sialic acid, alone or supplemented with either FBS or NAD and hemin.

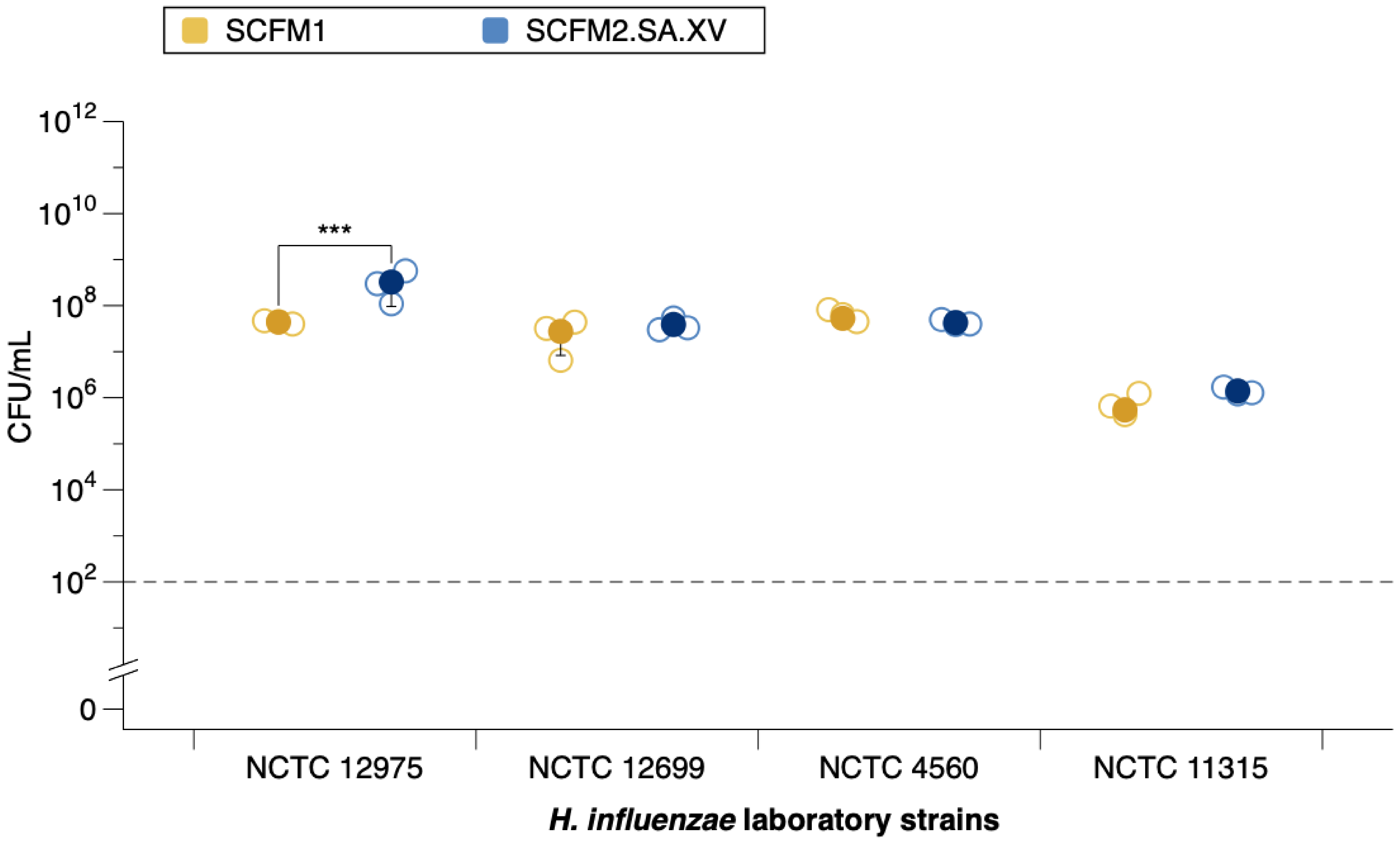

Supplementary Figure 2: CFU/mL of four laboratory *H. influenzae* strains following 24- hour growth in SCFM1 or SCFM2.SA.XV.
Mean and standard deviation was calculated from 3 replicates and shown by the solid circle. Significance was defined as p ≤ 0.05. Limit of detection is represented by the dotted line. There was a statistically significant difference in CFU between between media types (ANOVA: $F_{(1,16)} = 9.57$, $p = 0.007$), strains $F_{(3,16)} = 90.9136$, $p = <0.001$), and an interaction between media and strain $F_{(3,16)} = 4.1696$, $p = 0.0232$). Post-hoc analysis using estimated marginal means for pairwise comparisons highlighted which media*strain interactions were significant. There was an in the CFU of NCTC 12975 when grown in SCFM2.SA.XV compared to SCFM1 ($p = 0.0008$). The CFU of NCTC 11315 was significantly lower in both SCFM1 and SCFM2.SA.XV compared to the CFU of other three laboratory strains grown in these medias (all $p = <0.0001$). NCTC 12975 had a significantly higher CFU when grown in SCFM2.SA.XV (but not SCFM1) compared to NCTC 4560 ($p = 0.0034$), NCTC 12699 ($p = 0.0019$) and NCTC 11315 ($p = <0.0001$).

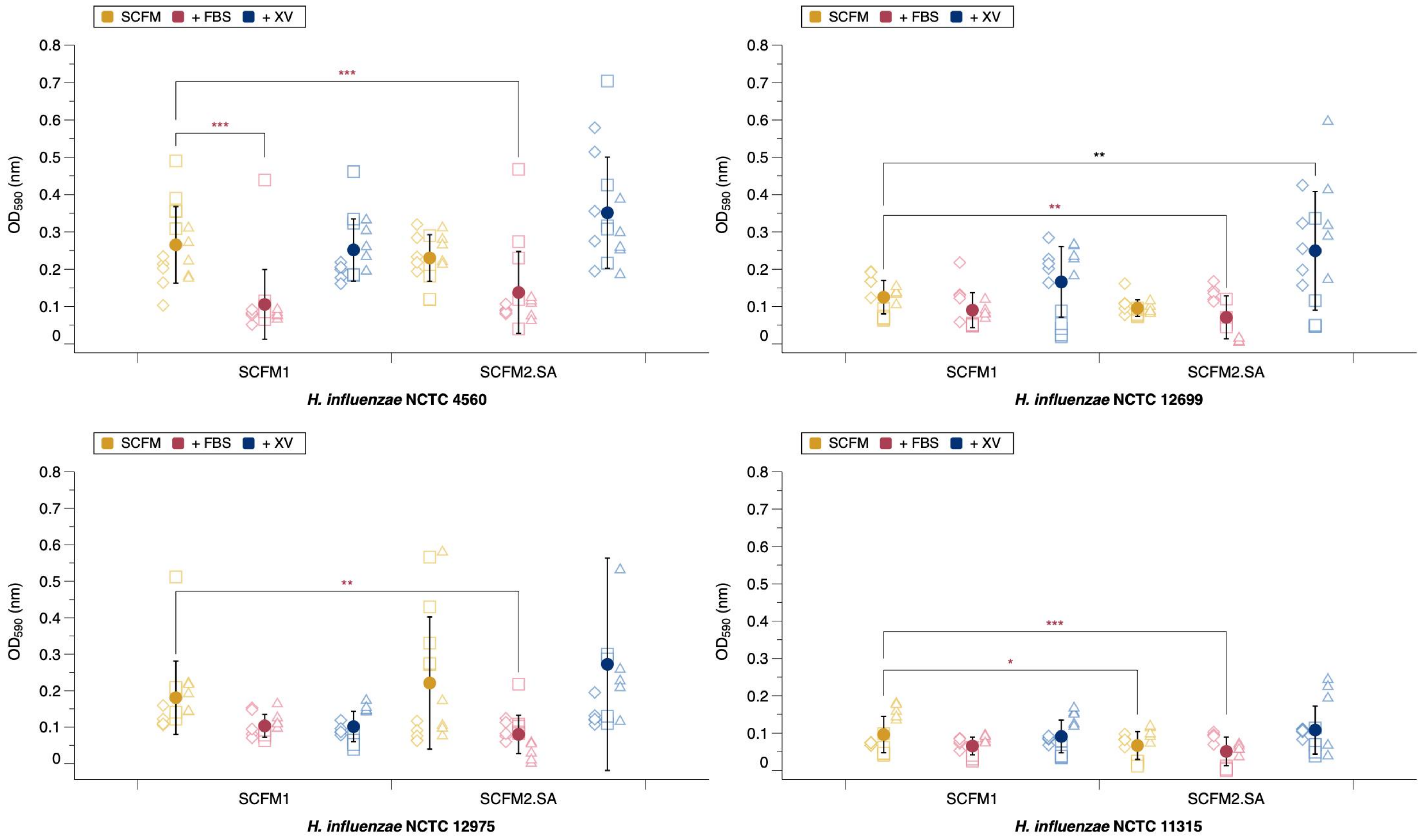

Supplementary Figure 3: 48-hour biofilm growth of four laboratory *H. influenzae* strains in varying SCFM conditions. Absorbance was measured at 590 nm. Mean and standard deviation was calculated from 3 independent experiments (demonstrated by different shapes) and shown by the solid circle. Significance was defined as $p \leq 0.05$. An ANOVA was run to test the effect of media variation on biofilm formation for each strain (NCTC 12975 $F_{5,82} = 6.78$, $p = < 0.001$, NCTC 12699 $F_{5,82} = 11.47$, $p = < 0.001$, NCTC 11315 $F_{5,82} = 10.21$, $p = < 0.001$, NCTC 4560 $F_{5,82} = 17.47$, $p < 0.001$). Post-hoc Dunnett analysis identified which media and strain comparisons resulted in a significant change in biofilm formation compared to SCFM1. A significant increase in biofilm growth was seen in NCTC 12699 ($p = 0.002$) when grown in SCFM2.SA.XV. Biofilm formation was significantly reduced in all strains when grown in SCFM2.SA.FBS (NCTC 12975 $p = 0.007$, NCTC 12699 $p = 0.008$, NCTC 11315 $p < 0.001$, NCTC 4560 $p < 0.001$), and a significant decrease was also seen in NCTC 4560 when grown in SCFM1.FBS ($p < 0.001$) and NCTC 11315 when grown in SCFM2.SA ($p = 0.027$).

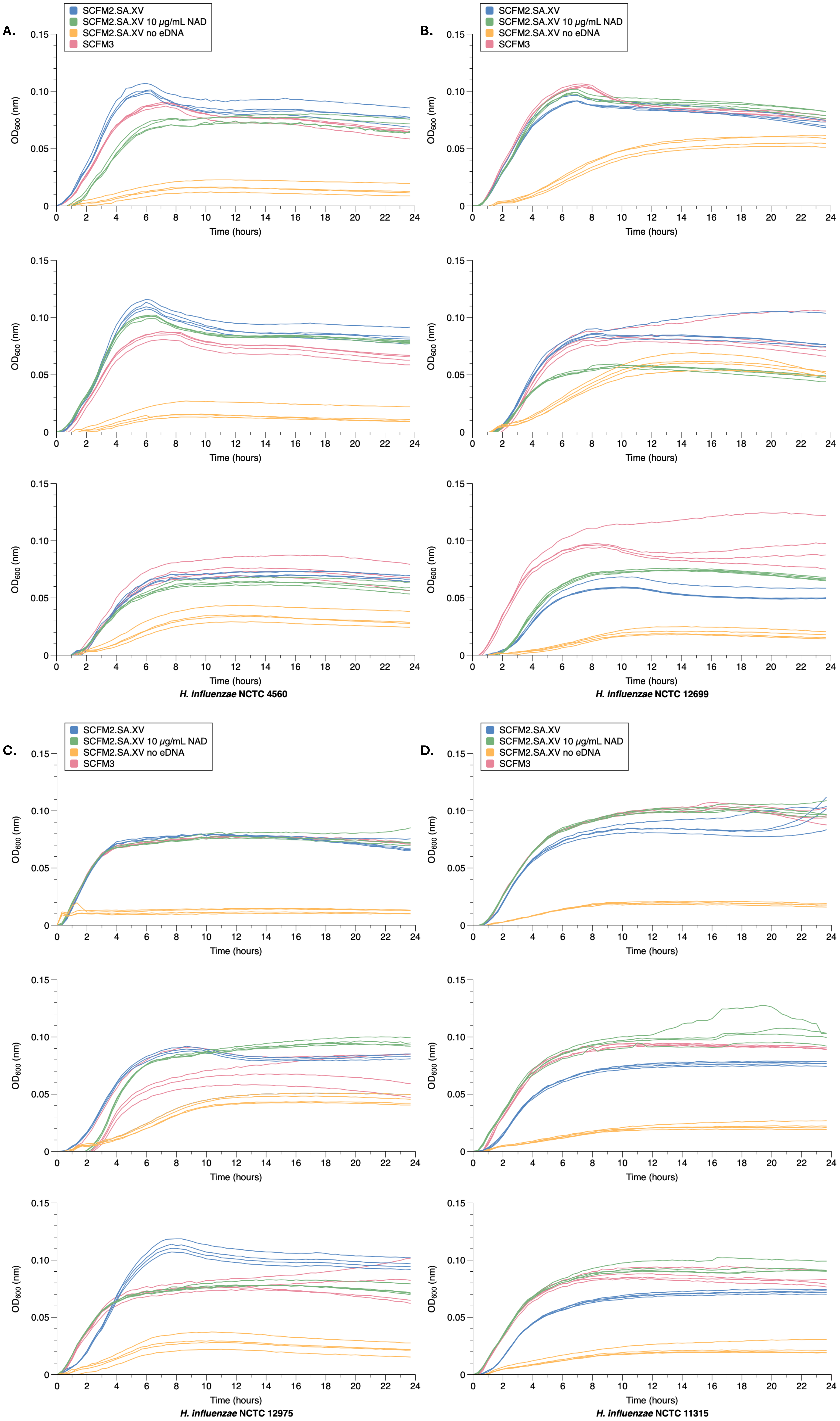

Supplementary Figure 4: Growth of *H. influenzae* laboratory strains in modifications of SCFM2.SA.XV over 24 hours. Three independent repeats were performed for each lab strain, **A.** *H. influenzae* NCTC 4560, **B.** *H. influenzae* NCTC 12699, **C.** *H. influenzae* NCTC 12975 and **D.** *H. influenzae* NCTC 11315 in 4 variants of media, SCFM2.SA.XV, SCFM2.SA.XV with 10 μg/mL of NAD, SCFM2.SA.XV with no eDNA and SCFM3.

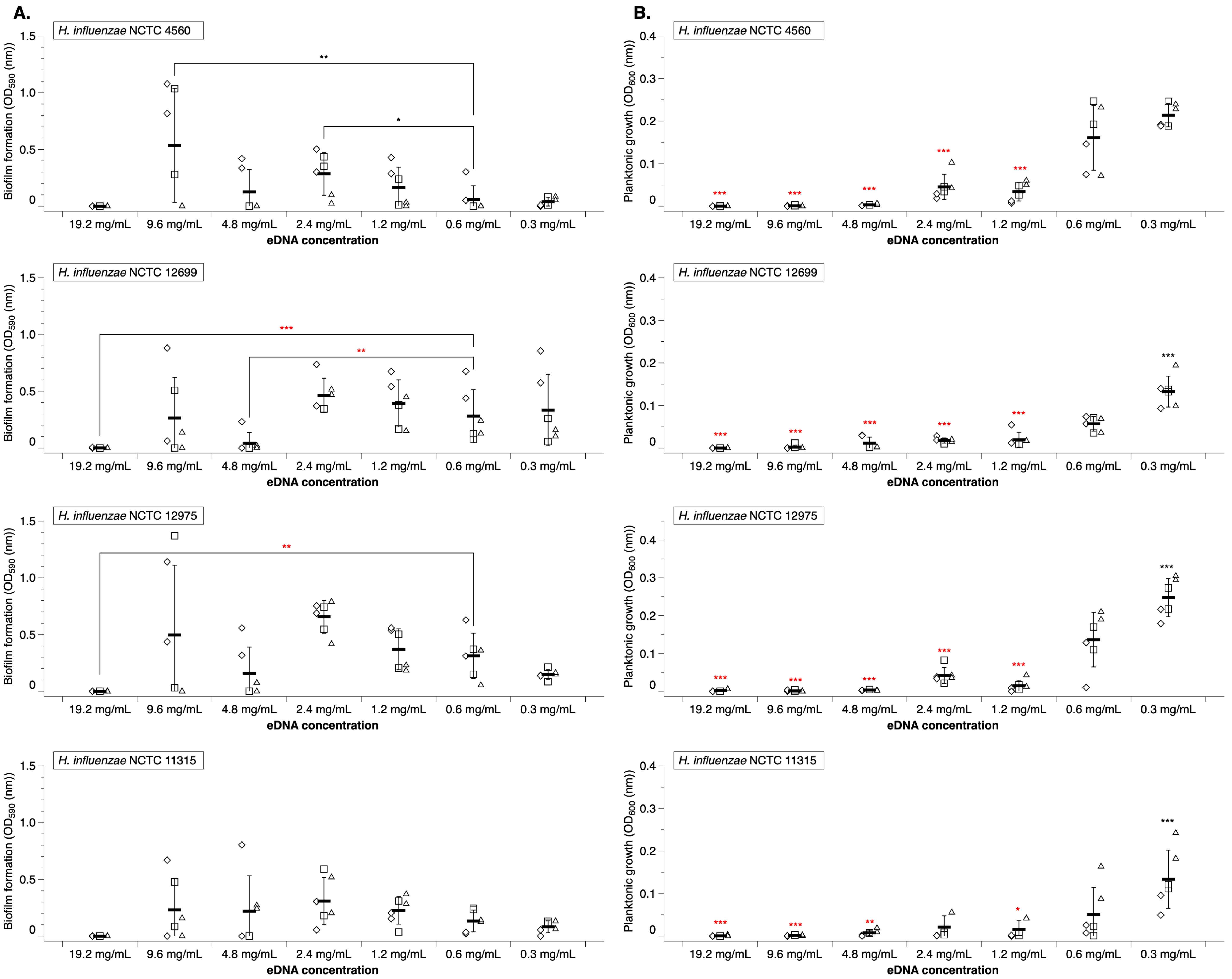

Supplementary Figure 5: 48-hour biofilm and planktonic growth of *H. influenzae* in SCFM2.SA.XV with varying extracellular DNA levels. A. Biofilm growth and **B**. Planktonic growth were measured by crystal violet staining and OD_{600} respectively. The mean and standard deviation are represented by the solid line and were calculated from 3 independent experiments (represented by different shapes). Significance was defined as $p \leq 0.05$. An ANOVA was run to test the effect of eDNA concentration on biofilm formation and planktonic growth. There was a significant effect of eDNA concentration on biofilm growth for *H. influenzae* NCTC 4560 ($F_{6,33}$ = 5.371 p = < 0.001), *H. influenzae* NCTC 12699 ($F_{6,33}$ = 11.24 p = < 0.001), *H. influenzae* NCTC 12975 ($F_{6,33}$ = 7.596 p = < 0.001) and *H. influenzae* NCTC 11315 ($F_{6,33}$ = 3.163 p = 0.015). There was also a significant effect on eDNA concentration on planktonic growth of *H. influenzae* NCTC 4560 ($F_{6,33}$ = 86.21 p = < 0.001), *H. influenzae* NCTC 12699 ($F_{6,33}$ = 50.784 p = < 0.001), *H. influenzae* NCTC 12975 ($F_{6,33}$ = 58.58 p = < 0.001) and *H. influenzae* NCTC 11315 ($F_{6,33}$ = 26.885 p = < 0.001). Post-hoc Dunnett analysis highlighted which concentrations of eDNA were significant compared to the standard of 0.06 mg/mL. A significant increase in biofilm formation was seen at 2.4 mg/mL (p = 0.032) and 9.6 mg/mL (p = 0.006) for *H. influenzae* NCTC 4560 compared to 0.06 mg/mL. A significant decrease was seen in biofilm formation at 4.8 mg/mL (p = 0.005) and 19.2 mg/mL (p =<0.001) for for *H. influenzae* NCTC 12699, and a decrease at 19.2 mg/mL (p = 0.002) for *H. influenzae* NCTC 12975 compared to 0.06 mg/mL. There was a significant decrease in planktonic growth at all concentrations compared to 0.06 mg/mL for all *H. influenzae* (p = <0.001) except *H. influenzae* NCTC 11315, and an increase seen at 0.03 mg/mL for all (p = <0.001) except *H. influenzae* NCTC 4560. There was a significant decrease in planktonic growth for *H. influenzae* NCTC 11315 at 1.2 mg/mL (p = 0.04), 4.8 mg/mL (p = 0.01), 9.6 mg/mL (p = < 0.001) and 19.2 mg/mL (p = <0.001).

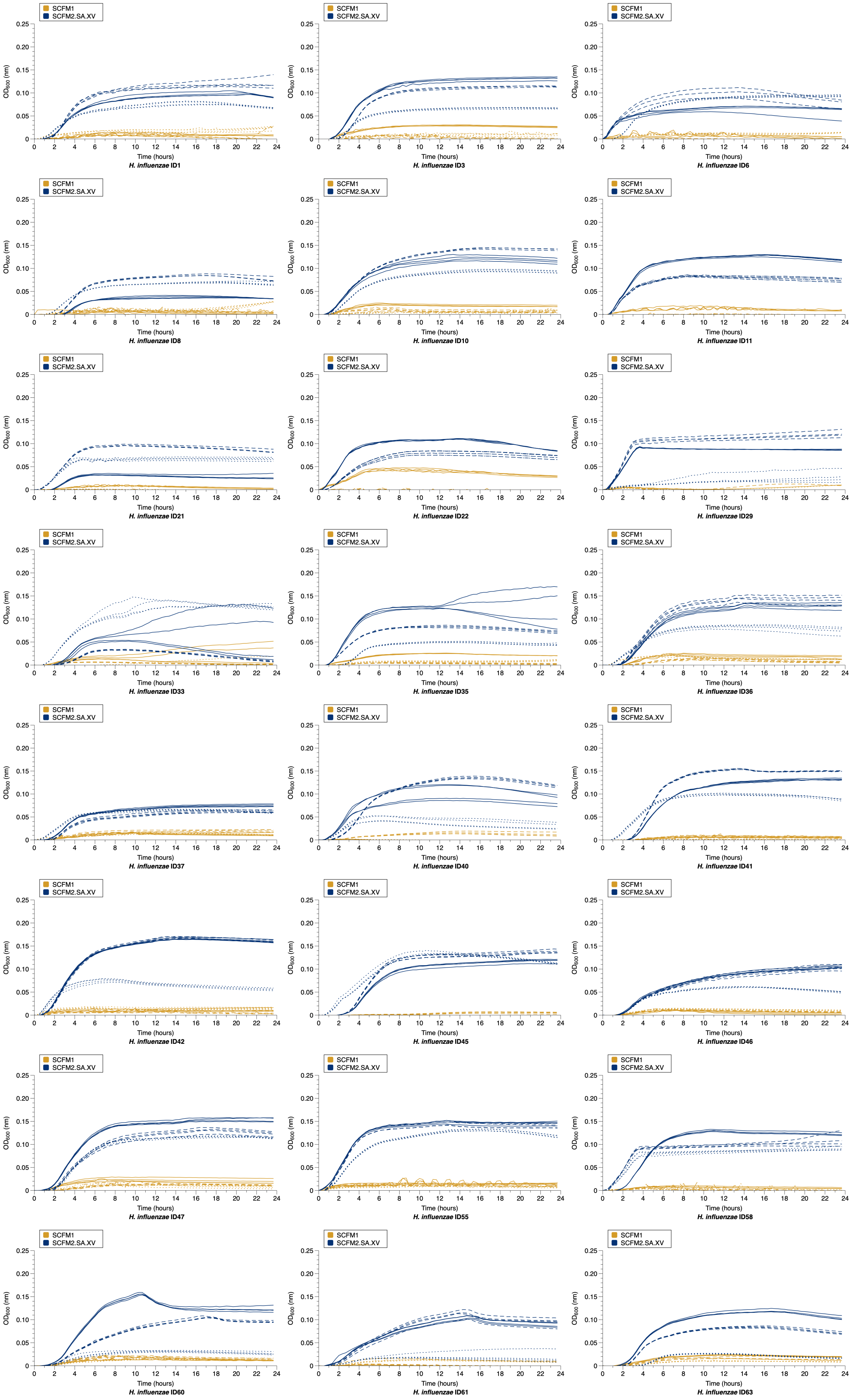

Supplementary Figure 6: Growth of *H. influenzae* cystic fibrosis clinical isolates in SCFM2.SA.XV and SCFM1 over 24 hours. Three independent repeats were performed for clinical isolates, with independent repeats shown by the solid line, dashed line and dotted line.

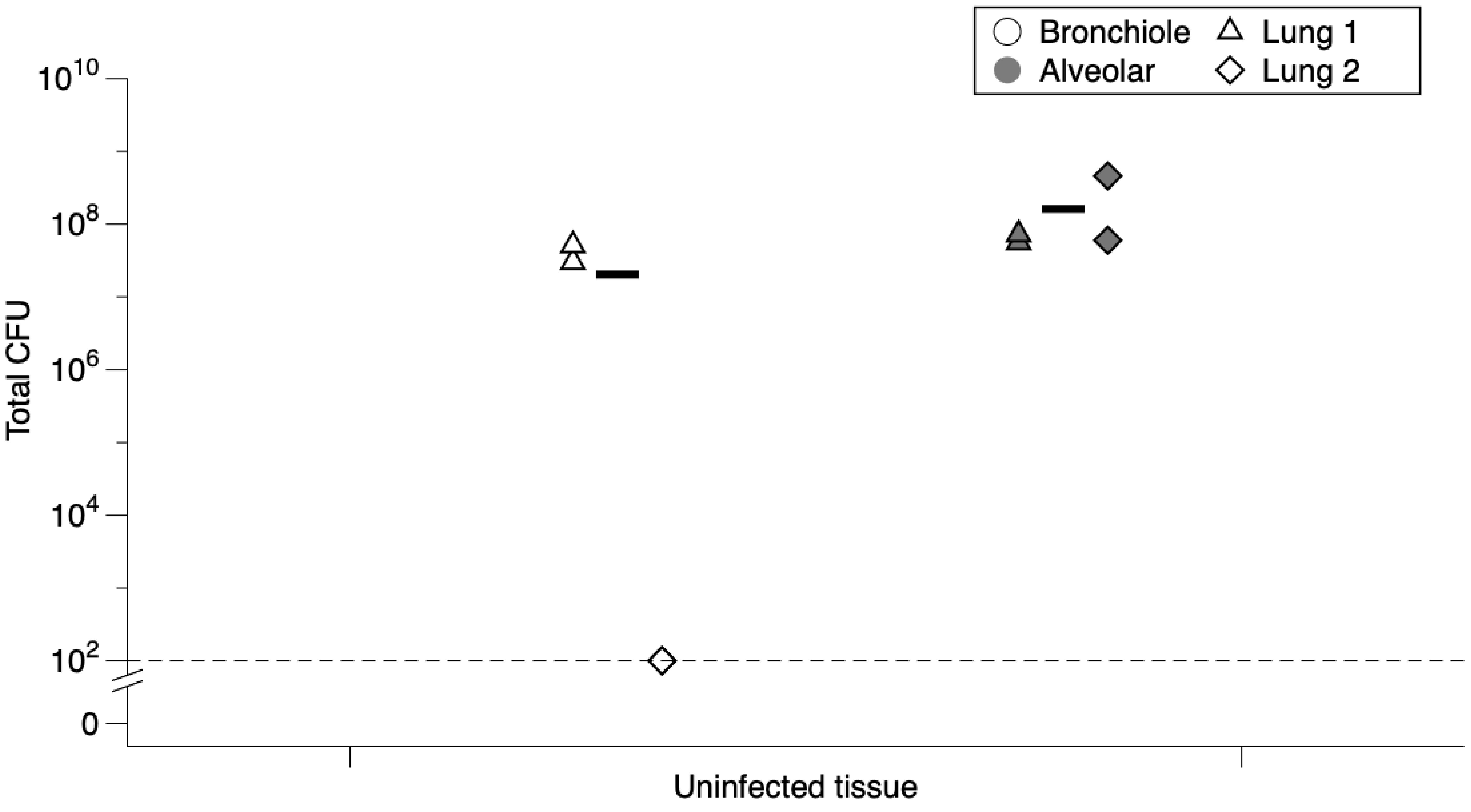

Supplementary Figure 7: Total CFU of endogenous bacteria on bronchiole and alveolar lung tissue. Means were calculated from 2 independent experiments (demonstrated by different shapes) and shown by the blue solid line.
